# The importance of the AMPK gamma 1 subunit in metformin suppression of liver glucose production

**DOI:** 10.1038/s41598-020-67030-5

**Published:** 2020-06-26

**Authors:** Hongying An, Yu Wang, Caolitao Qin, Mingsong Li, Akhil Maheshwari, Ling He

**Affiliations:** 1 Departments of Pediatrics, Johns Hopkins University School of Medicine, Baltimore, MD 21287 USA; 2 Pharmacology & Molecular Sciences, Johns Hopkins University School of Medicine, Baltimore, MD 21287 USA; 3 Department of Hepatology, Southern Medical University, Guangzhou, 510515 China

**Keywords:** Diabetes, Type 2 diabetes

## Abstract

Metformin has been used to treat patients with type 2 diabetes for over 60 years, however, its mechanism of action is still not completely understood. Our previous reports showed that high-fat-diet (HFD)-fed mice with liver-specific knockout of both AMPK catalytic α1 and α2 subunits exhibited significantly higher fasting blood glucose levels and produced more glucose than floxed AMPK catalytic α1 and α2 mice after long-term metformin treatment, and that metformin promotes the formation of the functional AMPK αβγ heterotrimeric complex. We tested the importance of each regulatory γ subunit isoform to metformin action in this current study. We found that depletion of γ1, but not γ2 or γ3, drastically reduced metformin activation of AMPK. HFD-fed mice with depletion of the γ1 subunit are resistant to metformin suppression of liver glucose production. Furthermore, we determined the role of each regulatory cystathionine-β-synthase (CBS) domain in the γ1 subunit in metformin action and found that deletion of either CBS1 or CBS4 negated metformin’s effect on AMPKα phosphorylation at T172 and suppression of glucose production in hepatocytes. Our data indicate that the γ1 subunit is required for metformin’s control of glucose metabolism in hepatocytes. Furthermore, in humans and animal models, metformin treatment leads to the loss of body weight, we found that the decrease in body weight gain in mice treated with metformin is not directly attributable to increased energy expenditure.

## Introduction

Diabetes affects at least 425 million people worldwide, with type 2 diabetes (T2D) accounting for more than 90% of diabetes cases. Metformin has been used clinically to treat patients with T2D since the 1950s^1,2^. Due to its efficacy in therapy and affordable price, metformin is now the most widely prescribed oral anti-diabetic agent worldwide, taken by over 150 million people annually^3^. In 2012, the American Diabetes Association and the European Association for the Study of Diabetes recommended metformin as the initial drug for treatment of patients with T2D^4^. In addition, metformin treatment has led to a reduction in cancer incidence^5–7^ and has extended the lifespan of patients with T2D^8,9^.

Metformin improves hyperglycemia in patients with T2D, an impact achieved mainly through the suppression of liver glucose production^10,11^. At the beginning of this century, metformin was reported to activate AMPK, a phylogenetically conserved serine/threonine kinase that presents in virtually all eukaryotes^12,13^. HFD-fed mice with liver-specific knockout of LKB1, an upstream kinase for AMPKα subunit phosphorylation at T172, are resistant to the effect of metformin on alleviation of hyperglycemia^14^. Our previous studies showed that pharmacological metformin concentration found in the portal vein (≤80 µM) is unable to suppress cAMP- or glucagon-stimulated glucose production in primary hepatocytes with loss of both AMPK catalytic α subunits^15^. HFD-fed mice with liver-specific knockout of both AMPK catalytic α1 and α2 subunits produced significantly more glucose compared to floxed AMPKα1 and α2 mice after long-term treatment with a clinically relevant metformin dose (50 mg/kg/day)^16^. These data demonstrate that liver AMPKα1 and α2 subunits have important roles in metformin’s control of glucose metabolism and improvement of hyperglycemia in HFD-fed mice. Furthermore, activation of AMPK by metformin augments the phosphorylation of CREB-binding protein (CBP) at S436 via atypical protein kinase ι/λ, resulting in the disassembly of the CREB co-activator complex, inhibition of gluconeogenic gene expression and a reduction in glucose production^17,18^.

Functional AMPK is a heterotrimeric complex consisting of a catalytic α subunit, scaffold protein β subunit, and regulatory γ subunit; each subunit exists as multiple isoforms (α1, α2, β1, β2, γ1, γ2, γ3) and is encoded by separate genes^19–21^. The regulatory γ subunit contains a tandem of four cystathionine-β-synthase (CBS) domains that serve as the adenine nucleotide-binding region^22^. Phosphorylation by upstream kinases of the AMPKα subunit at T172, a conserved phosphorylation site in the α subunit, leads to an over 100-fold increase in kinase activity. When the cellular energy status falls, an increased AMP/ATP or ADP/ATP ratio leads to AMP or ADP binding to the γ subunit, resulting in an allosteric change in the AMPK complex, and augmenting the phosphorylation of α subunit at T172 either by an upstream kinase or by preventing dephosphorylation by a protein phosphatase^23–25^. The isoforms of each subunit could form 12 different AMPK heterotrimeric complexes, each one having a distinct function^26^. Since the regulatory γ subunit plays a critical role in the activation of the catalytic α subunit and AMPK heterotrimeric complexes containing different γ subunit isoforms are regulated differently^26^, we therefore determined the importance of each γ subunit isoform and individual CBS domain in the γ subunit to metformin action in this current study. These data provide important new insights into the mechanisms of metformin action.

## Results

### Determination of each γ subunit isoform in metformin-mediated AMPK activation

**Figure 1:**
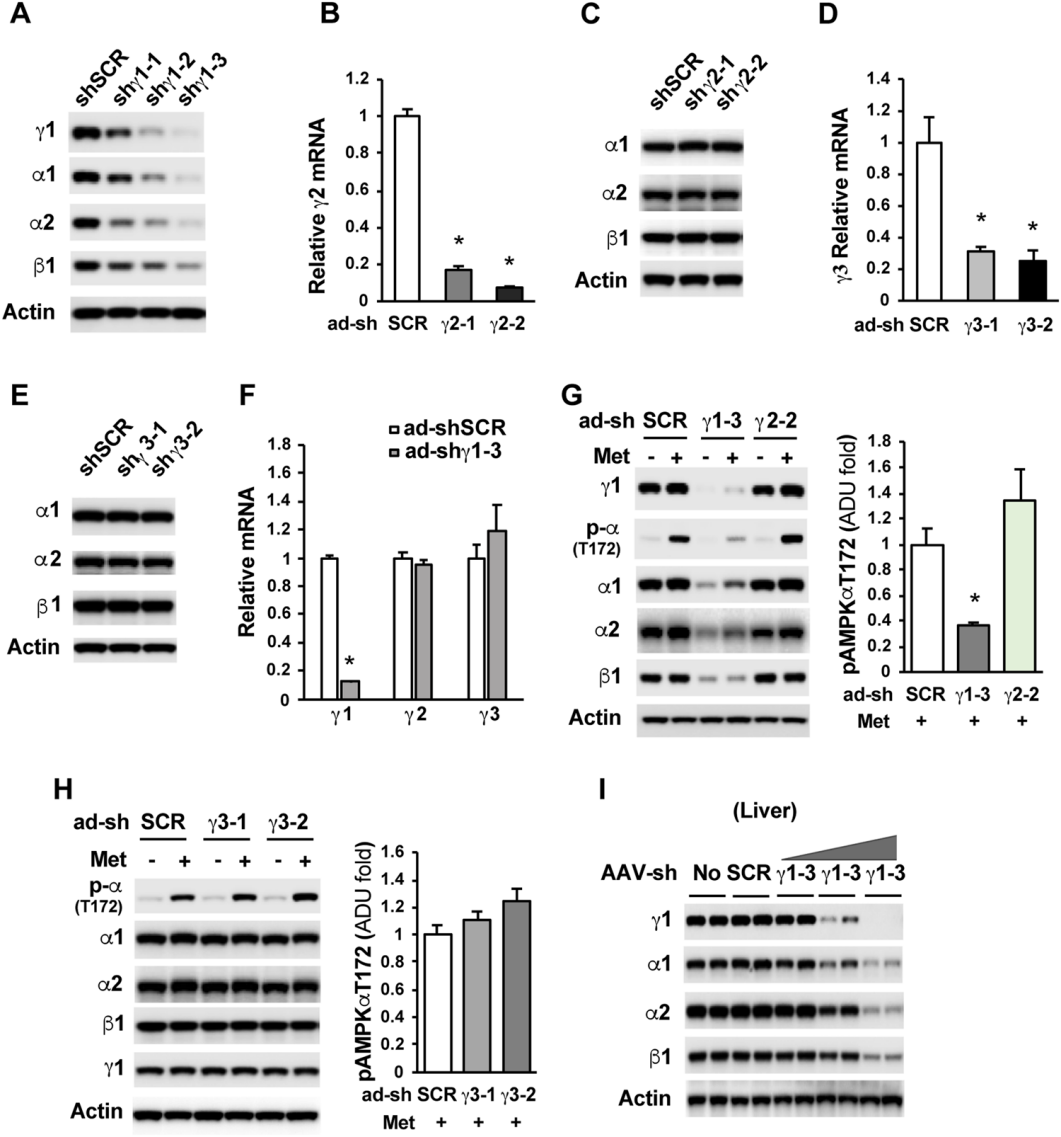
The importance of  γ subunit in AMPKα activation by metformin. (**A)** Three sets of adenoviral shRNAs of AMPKγ1 (shγ1-1, shγ1-2, and shγ1-3) were added to Hepa1-6 cells for 48 h. The shγ1-3 shRNA had the strongest effect, therefore, was used to deplete AMPKγ1 in the following experiments. (**B,C**) The mRNA levels of AMPKγ2 in Hepa1-6 cells treated with two sets of AMPKγ2 shRNAs for 48 h (**B**). Depletion of AMPKγ2 had no effect on the protein levels of AMPKα1, α2, and β1 **(C)**. (**D,E**) The mRNA levels of AMPKγ3 in Hepa1-6 cells treated with two sets of AMPKγ3 shRNAs for 48 h (**D**). Depletion of AMPKγ3 had no effect on the protein levels of AMPKα1, α2, and β1 (**E**). (**F**) Hepa1-6 cells were treated with shγ1-3 shRNA as in (**A**) (n = 3). (**G**) 24 h after the addition of adenoviral shRNAs, Hepa1-6 cells were treated with 2 mM metformin for 16 h. Right panel, densitometric analysis of AMPKα phosphorylation at T172 (n = 3). (**H**) Two sets of AMPKγ3 adenoviral (ad-shγ3-1 and ad-shγ3-2) shRNAs were added to Hepa1–6 cells for 24 h followed by the addition of 2 mM metformin for 16 h. Right panel, densitometric analysis of AMPKα phosphorylation at T172 (n = 3). (**I**) C57BL6 mice were injected with AAV8 scrambled shRNA (1X10^12^ GC/mouse), or AAV8 AMPKγ1 shRNA at 1X10^10^ GC, 1X10^11^ GC, and 1X10^12^ GC per mouse. Liver samples were collected 2 weeks after the viral injection. Each lane represents an individual mouse liver sample. *p < 0.05, Student’s t-test.

To date, it has been remained undecided whether each γ subunit isoform has a role in AMPKα activation by metformin in cultured cells or *in vivo*. To assess the role of each γ subunit isoform in metformin-mediated AMPKα activation, we first generated adenoviral shRNAs to deplete each γ subunit isoform in hepatoma Hepa1–6 cells. We observed the greatest depletion of γ1 protein levels when adenoviral shγ1–3 was used (Fig. 1A). Therefore, this set of shRNA was employed in our evaluation of γ1 subunit isoform in metformin-mediated AMPKα activation (Fig. 1A). Since we do not have reliable antibodies against γ2 and γ3 subunit isoforms, we determined the mRNA levels of γ2 and γ3 subunit isoforms in Hepa1–6 cells treated with two sets of adenoviral shRNAs for each subunit isoform and found that these shRNAs effectively depleted their target genes (Fig. 1B,D). Depletion of the γ1 subunit isoform had no effect on the mRNA levels of γ2 and γ3 subunit isoforms (Fig. 1F), and depletion of either γ2 or γ3 subunit isoform also did not affect the expression of the γ1 subunit isoform (Fig. 1G,H), indicating the high specificity of each shRNA. Using these adenoviral shRNAs, we depleted γ1, γ2, and γ3 in Hepa1–6 cells, treated these cells with metformin, and found that depletion of γ1 almost completely abolished the phosphorylation of AMPKα at T172 by metformin. In contrast, depletion of either γ2 or γ3 did not reduce AMPKα phosphorylation at T172 by metformin (Fig. 1G,H). Depletion of γ1, but not γ2 or γ3, also significantly decreased the protein levels of the AMPKα1, α2, and β1 subunits in Hepa 1–6 cells and in the liver of mice (Fig. 1A,C,E,G–I). Of note, depletion of either γ2 or γ3 had not significant effect on the mRNA levels of γ1 as well (data not shown).

**Figure 2:**
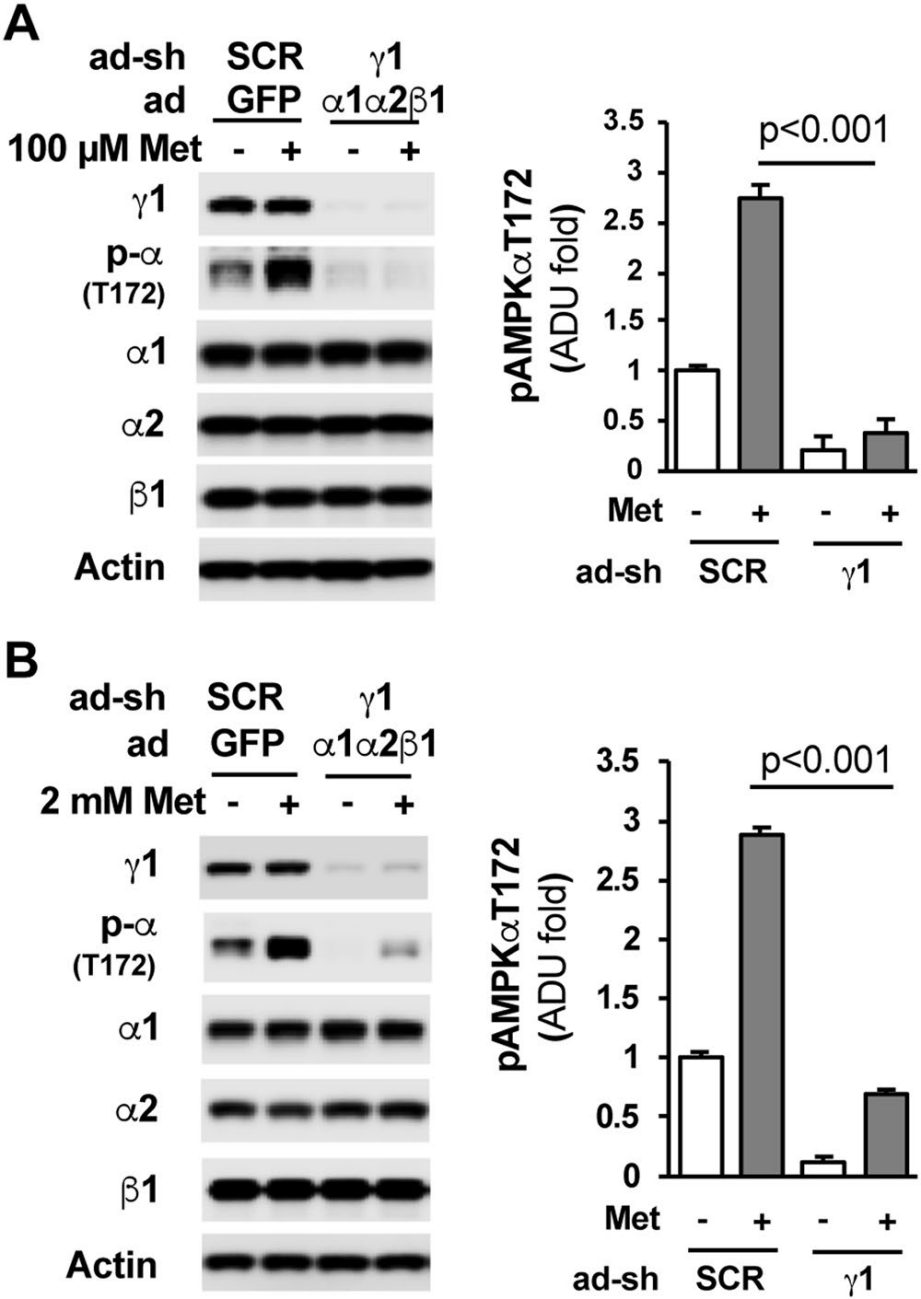
Depletion of the γ1 subunit abolished metformin-mediated AMPKα activation. (**A**) 24 h after the addition of adenoviral shRNAs for scrambled control and AMPKγ1 plus adenoviral expression vectors for AMPK α1, α2, and β1, Hepa1-6 cells were treated with 100 µM metformin for 16 h, and then, medium was changed to FBS-free DMEM, and 100 µM metformin was added; cells were harvested 4 h later. Right panel, densitometric analysis of AMPKα phosphorylation at T172 (n = 3). (**B**) 24 h after the addition of adenoviral shRNAs for scrambled control and AMPKγ1 plus adenoviral expression vectors for AMPK α1, α2, and β1, Hepa1-6 cells were treated with 2 mM metformin for 16 h. Right panel, densitometric analysis of AMPKα phosphorylation at T172 (n = 3). *p < 0.05, Student’s t-test.

To eliminate confounding reductions in the AMPKα1, α2, and β1 subunits, we used adenoviral expression vectors to express similar protein levels of AMPKα1, α2, and β1 subunits in Hepa1–6 cells treated with adenoviral-γ1-shRNA as in Hepa1–6 cells treated adenoviral-scrambled shRNA control. We found that depletion of γ1 abolished AMPKα phosphorylation at T172 by low concentrations (100 µM) and high concentrations (2 mM) of metformin (Fig. 2A,B). The above data demonstrate that among the three regulatory γ subunit isoforms, γ1 is required for AMPK activation by metformin in hepatocytes.

### Depletion of the γ1 subunit by AAV-shRNA markedly increased liver glucose production

**Figure 3:**
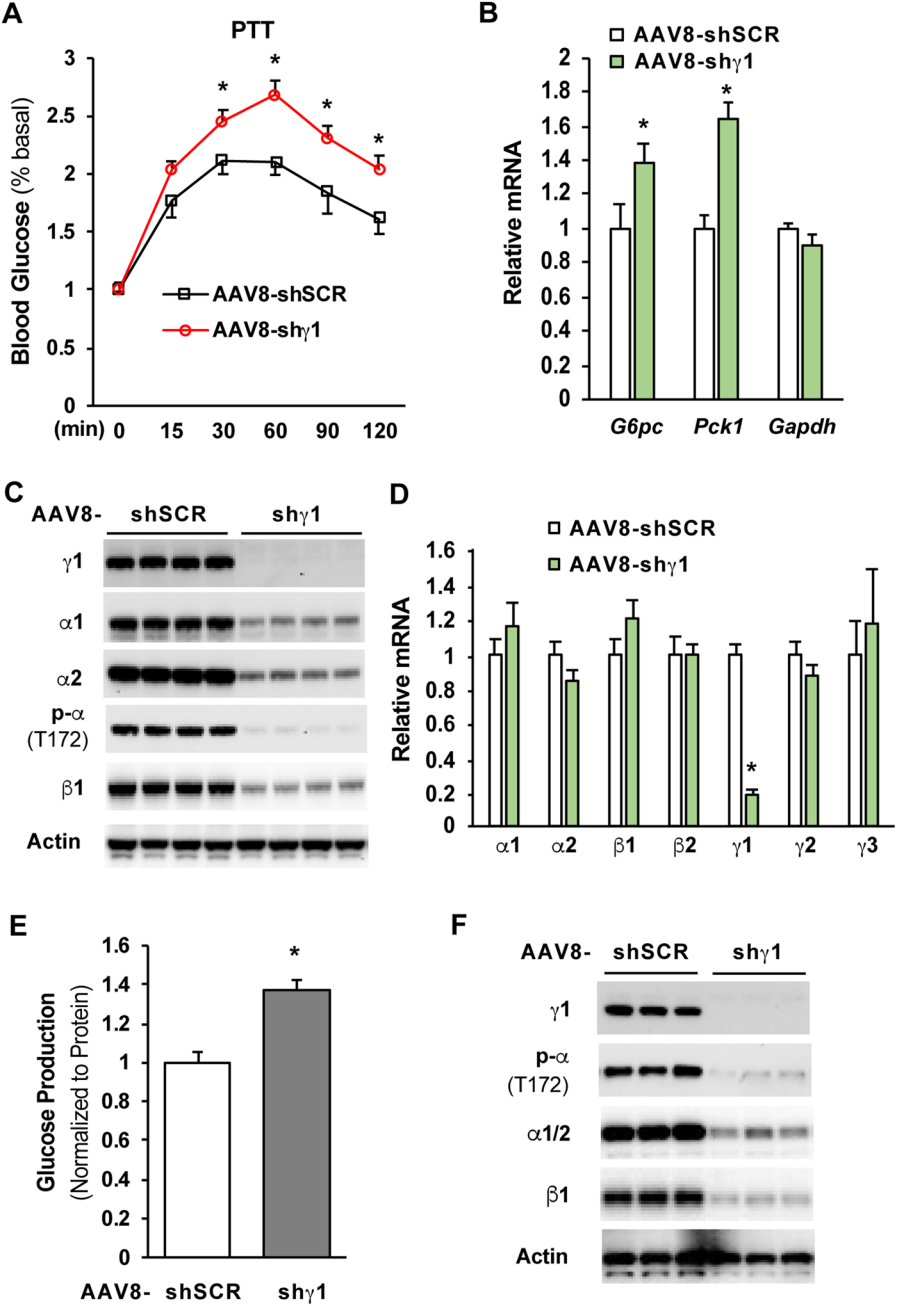
Depletion of the γ1 subunit by AAV-shRNA increased liver glucose production in HFD-fed mice treated with metformin. (**A–D**) C57BL6/J mice were fed an HFD for 4 weeks, and then mice were injected with AAV8 scrambled shRNA or γlshRNA vectors (1X10^12^ GC per mouse) through jugular vein. After 3 weeks of treatment with metformin (50 mg/kg/day), a pyruvate tolerance test (6 h fast, 1.5 mg/kg) was conducted (n = 5/group) (**A**), and liver tissues were collected, followed by determination of the mRNA levels of the gluconeogenic enzyme gene (**B**) and the protein (**C**) and mRNA (**D**) levels of AMPK subunits in the liver. (**E,F**) Primary hepatocytes were prepared from mice treated with AAV-shRNAs and metformin as in (**A**), glucose production assay was conducted 48 h after the planting (n = 3)(**E**). Indicated proteins were determined in the primary hepatocytes (**F**). *p < 0.05, Student’s t-test.

Since metformin improves hyperglycemia in T2D mainly through suppression of liver glucose production^10,11^, we assessed the importance of the γ1 subunit in metformin suppression of liver glucose production. C57BL6 mice were fed an HFD for 4 weeks to induce insulin resistance^27^, followed by injection of AAV8-scrambled shRNA or -γ1shRNA to deplete the γ1 subunit in the liver of HFD-fed mice. Both groups of mice were then given a clinically relevant metformin dose (50 mg/kg/day) through drinking water for 3 weeks^15,16^. HFD-fed mice with depletion of liver AMPKγ1 subunit exhibited significantly higher blood glucose levels in a pyruvate tolerance test and elevated mRNA levels of *Pck1* and *G6pc* in the liver (Fig. 3A,B). Depletion of the γ1 subunit led to significant reductions in α1, α2, and β1 subunits in the liver (Fig. 3C), which occurred at the posttranscriptional level because their mRNA levels were not significantly affected (Fig. 3D). Additionally, primary hepatocytes prepared from metformin-treated mice with depletion of liver γ1 subunit produced significantly more glucose compared to primary hepatocytes prepared from metformin-treated mice without depletion of liver γ1 subunit (Fig. 3E,F).

**Figure 4:**
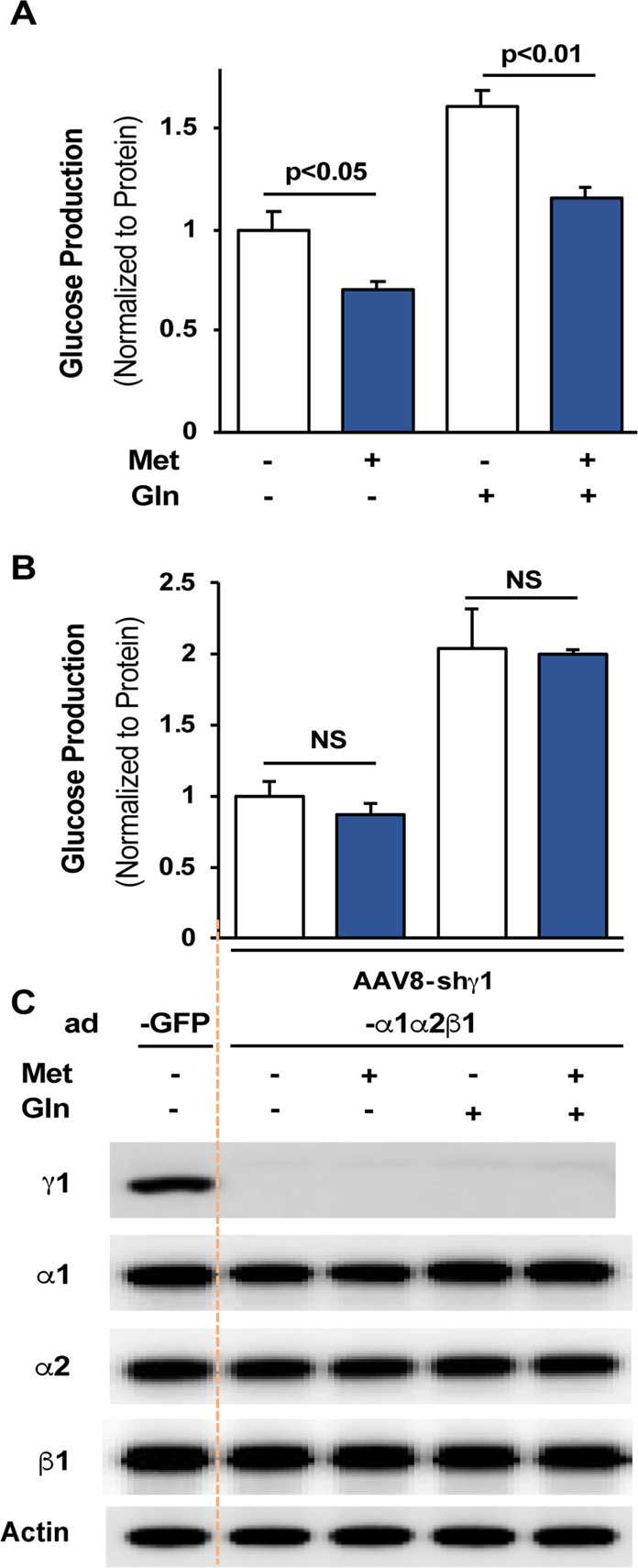
The γ1 subunit is required for metformin suppression of glucose production in primary hepatocytes. (**A***)* Primary hepatocytes prepared from mice injected with AAV8 null vector (18 days) were treated with 100 µM metformin for 16 h, and then, medium was changed to FBS-free DMEM, and 100 µM metformin was added for 3 h, followed by glucose production medium supplemented with metformin and 10 nM glucagon for another 3 h. (**B,C**) 6 h after the planting of primary hepatocytes prepared from mice injected with AAV γ1shRNA (18 days), adenoviral expression vectors for AMPKα1, α2, and β1 were added. Primary hepatocytes were treated as in (**A**). Glucose was measured in the medium (**B***)* (n = 3), and cellular lysates were subjected to immunoblots (**C**). N.S., not significant.

To accurately define the role of the γ1 subunit in metformin action without confounding decreases in endogenous protein levels of the α1, α2, and β1 subunits in hepatocytes (Fig. 3C,F), we prepared primary hepatocytes from mice with depletion of liver AMPKγ1 and used adenoviral expression vectors to express comparable protein levels of α1, α2, and β1 subunits to their corresponding endogenous levels in primary hepatocytes prepared from mice without depletion of liver γ1 subunit. In glucose production assays, metformin significantly suppressed glucagon-stimulated production in primary hepatocytes prepared from mice treated with AAV8-scrambled shRNA (Fig. 4A); in contrast, metformin failed to suppress glucagon-stimulated production in primary hepatocytes with depletion of the γ1 subunit (Fig. 4B,C).

### The importance of the CBS domain in the γ1 subunit in metformin action

**Figure 5:**
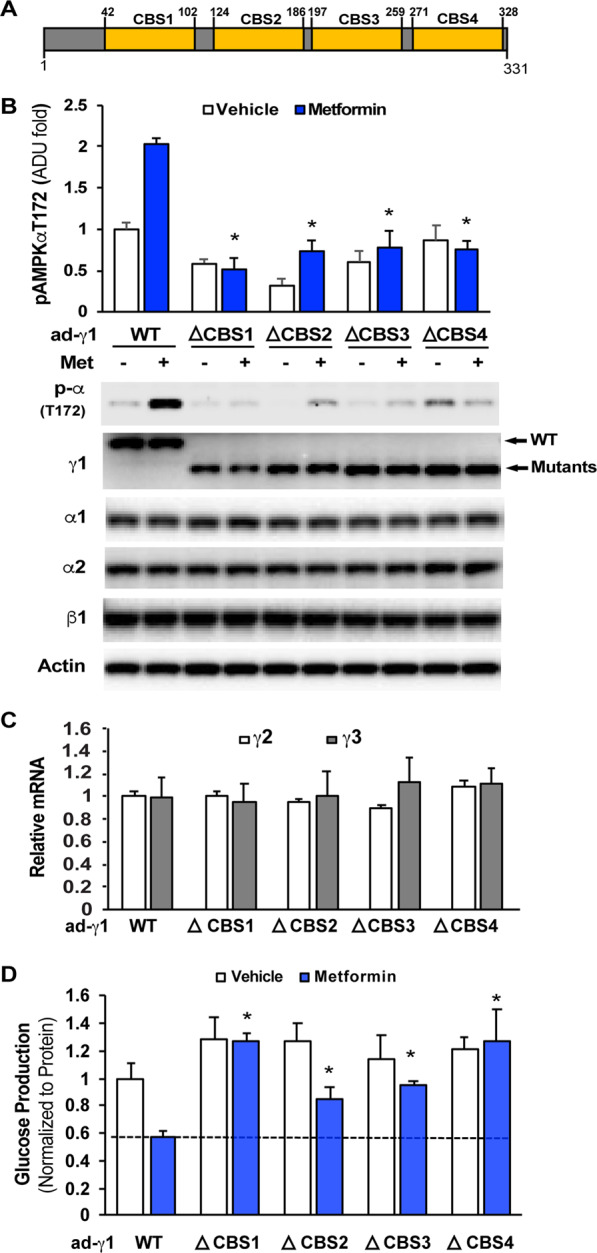
The γ1 subunit is essential for metformin activation of AMPK. (**A**) The schematic annotation of 4 CBS domains in the γ1 subunit. (**B**) 24 h after the addition of adenoviruses, Hepa1-6 cells were treated with 100 µM metformin for 16 h, and then medium was changed to FBS-free DMEM, and 100 µM metformin was added for 3 h (n = 3).(**C**) Hepa1-6 cells were treated with adenoviruses for 48 (n = 3). (**D**) After 6 h of planting, adenoviruses were added to the primary hepatocytes for 2 h, followed by treatment with 100 µM metformin for 40 h, and then medium was changed to FBS-free DMEM, and 100 µM metformin was added for 3 h (n = 3). *p < 0.05.

The four CBS domains in the γ subunit are the binding sites for the regulatory nucleotides AMP, ADP, and ATP^22^. Our previous study showed that metformin can promote the formation of the AMPKαβγ heterotrimeric complex^28^. We tested further the importance of these CBS domains in the γ1 subunit in metformin activation of AMPKα by generating four adenoviral expression vectors such that individual CBS domains were  deleted in a FLAG-tagged γ1 subunit (Fig. 5A). Using these expression vectors, we expressed comparable amounts of γ1-WT and its mutants in Hepa1–6 cells and treated these cells with metformin. As shown in Fig. 5B, deletion of each CBS domain significantly decreased basal and metformin-stimulated AMPKα phosphorylation at T172 (Fig. 5B). In particular, deletion of CBS1 and CBS4 completely abolished metformin effect on AMPKα phosphorylation at T172. Deletion of each CBS domain had not significantly impact on the gene expression of γ2 or γ3 subunit isoform (Fig. 5C). The above data substantiate further the importance of γ1 subunit isoform in metformin-mediated AMPK activation. Metformin treatment significantly suppressed glucose production in primary hepatocytes with expression of γ1-WT; in contrast, primary hepatocytes with expression of a γ1 mutant produced more glucose when treated with metformin. Specifically, primary hepatocytes with expression of a γ1 subunit with deletion of either the CBS1 or CBS4 domain produced significantly more glucose than primary hepatocytes with expression of γ1-WT that were treated with vehicle (Fig. 5D), suggesting that these CBS domains are required for metformin action.

### Effects of metformin on energy expenditure

**Figure 6:**
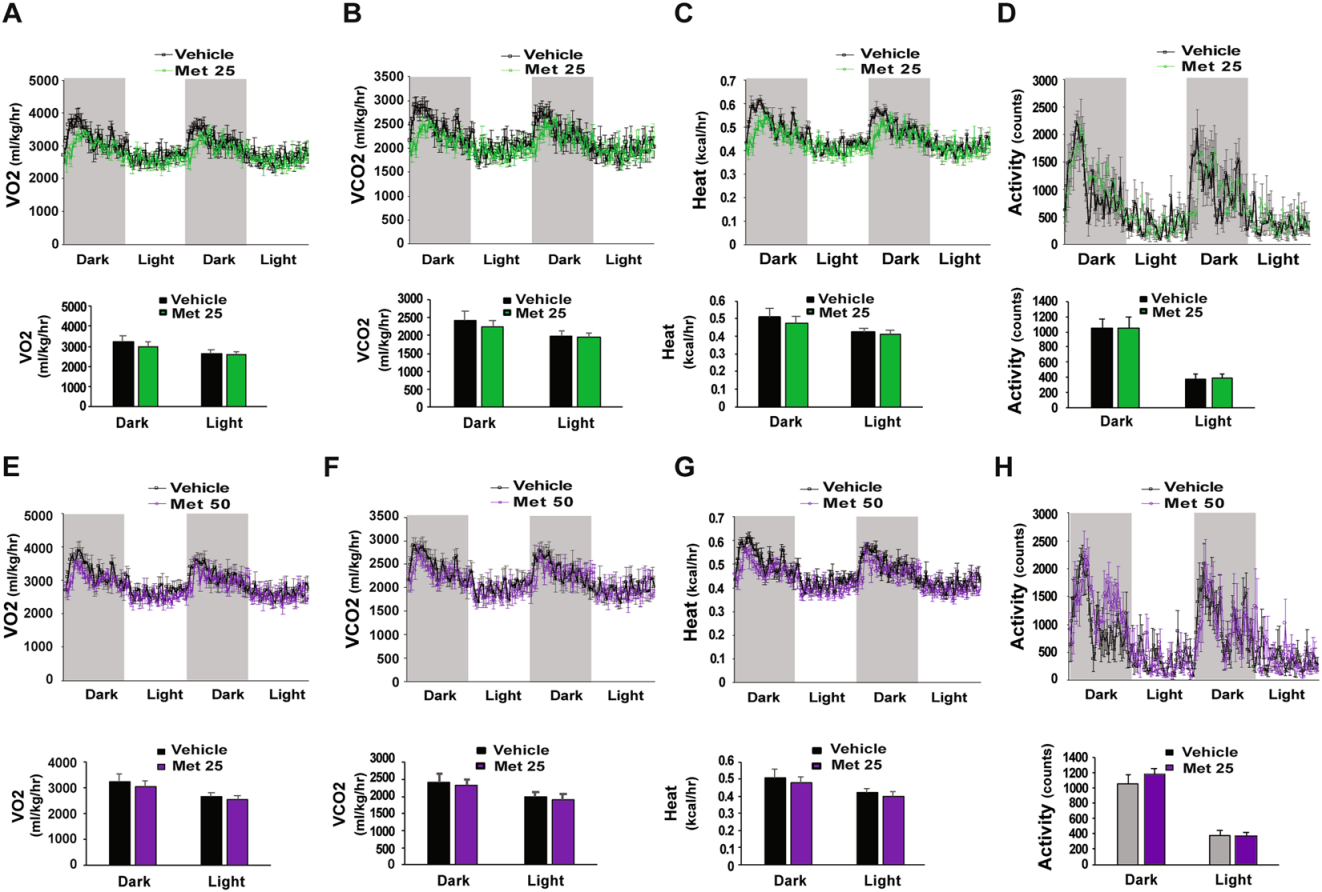
Energy expenditure in HFD-fed mice treated with metformin. (**A–D**) Oxygen consumption (**A**), CO2 production (**B**), heat production (**C**), and activity (**D**) in mice fed an HFD for 4 weeks and then treated with 25 mg/kg of metformin for another 5 weeks (n = 6/group). (**E–H**) Oxygen consumption (**E**), CO2 production (**F**), heat production (**G**), and activity (**H**) in mice fed an HFD for 4 weeks and treated with 50 mg/kg of metformin for another 5 weeks (n = 6/group).

Clinically, patients with T2D treated with metformin have body weight loss^29,30^. Our previous report showed that treatment with clinically relevant doses of metformin at 25 or 50 mg/kg/day led to reductions of body weight gain by 46% in HFD-fed mice^16^. To test whether the decrease in body weight gain in mice treated with metformin^16^ was due to changes in energy expenditure, we conducted indirect calorimetry analyses on HFD-fed mice treated with two doses of metformin (25, 50 mg/kg/day) to examine whole body energy expenditure. Real-time monitoring showed that oxygen consumption (VO_2_) was not significantly affected by metformin in the tested doses (Fig. 6A,E). After separation of data into dark and light cycle, metformin still had no significant effect on VO_2_ (Fig. 6A,E, lower panels). Similarly, metformin had no significant effects on VCO_2_ (Fig. 6B,F). In addition, metformin did not significantly change calculated body heat and activity (Fig. 6C,D,G,H). Overall, we found that metformin-treated mice tended to have relatively lower VO_2_, VCO_2_, and heat generation, even though these data did not reach statistical significance (Fig. 6). Therefore, the decrease in body weight gain in mice treated with metformin is not directly attributable to increased energy expenditure. In agreement with other reports in human subjects^29,30^, we found that treatment with metformin (50 mg/kg/day) reduced the food consumption by 35% in HFD-fed mice^16^, thus, the observed decrease in body weight gain may be due to reduced food intake, which is through the induction of GDF15 by metformin^31–33^. Interestingly, we found that metformin treatment increased daily water consumption, mice treated with metformin at 0, 25, 50 mg/kg/dy of metformin drank 2.75 ± 0.05, 3.60 ± 0.07, 3.85 ± 0.14 mL water, respectively.

## Discussion

Metformin is a first-line antidiabetic drug and functions mainly by improving patients’ hyperglycemia through suppression of liver glucose production^10,11^. However, its mechanism of action is still not well understood and remains controversial. It has been proposed that the principal mechanism of metformin action is through an AMPK-independent pathway^34,35^. To test whether AMPK has a role in metformin suppression of glucose production in hepatocytes, we employed adenoviral shRNA to deplete both AMPK catalytic α1 and α2 subunits in primary hepatocytes and found that pharmacological concentrations of metformin were unable to suppress glucose production^15^. Recently, we reported that HFD-fed mice with embryonic liver-specific knockout of both AMPKα1 and α2 subunits exhibited significantly higher fasting blood glucose levels and produced more glucose than double floxed AMPKα1 and α2 mice after long-term metformin treatment^16^. Furthermore, primary hepatocytes prepared from adult mice with transition knockout of liver AMPKα1 and α2 subunits and treated with metformin for 3 weeks produced significantly more glucose than primary hepatocytes prepared from metformin-treated adult mice without transition knockout of liver AMPKα1 and α2 subunits^16^. These studies clearly demonstrate that the AMPK catalytic α1 and α2 subunits are required for metformin’s suppression of liver glucose production and improvement of hyperglycemia in HFD-fed mice.

We previously found that AMPK subunits are not equally expressed in hepatocytes, and metformin can promote the formation of the AMPK heterotrimeric αβγ complex, resulting in increased AMPKα phosphorylation at T172 by the upstream kinase LKB1^28^. We, therefore, examined the role of the regulatory γ subunit in metformin-mediated AMPKα phosphorylation at T172 and suppression of liver glucose production in the current study. Since there are three regulatory γ subunit isoforms exist in hepatocytes and AMPK heterotrimeric complexes containing different γ subunit isoforms are regulated differently^26^, we generated shRNA to deplete each γ subunit isoform and found that depletion of only γ1 abolished AMPKα activation by metformin. These data are reminiscent of the structural differences between the γ subunit isoforms. In N-terminal regions, both γ2 and γ3 have an extra region that contains 240 and 150 a.a., respectively; and that extra region is missing in γ1^36,37^. Depletion of the γ2 or γ3 subunit isoform results an tendency toward increased AMPKα phosphorylation at T172, making it tempting to speculate that γ2 or γ3 might have a negative effect on metformin activation of AMPKα by competing for the catalytic α subunit with γ1. In agreement with a previous report^38^, our results show that loss of γ1 led to the reductions in α1, α2, and β1 subunits in hepatocytes, occurring at the posttranscriptional levels. On the other hand, we found that the loss of both AMPKα catalytic subunits also led to the reduction of β1 and γ1 subunits^16^, these data suggest that the formation of AMPKαβγ heterotrimeric complex can resist these proteins’ degradation.

To determine the domain(s) in the γ1 subunit important for metformin interactions, we found that deletion of individual CBS domain significantly reduced AMPKα phosphorylation at T172. Since AMP and ADP bind to the CBS1 or CBS3 domain to activate AMPK^22^, and a pharmacological concentration of metformin does not alter the AMP or ADP levels^15^, this suggests that the intact structure of the γ1 subunit is important for maintaining AMPK activity. However, individual CBS domains in the γ1 subunit do not contribute equally to the activation of AMPKα by metformin. Deletion of either the CBS1 or CBS4 domain of the γ1 subunit completely abolished metformin-mediated phosphorylation of AMPKα at T172. In contrast, after deletion of either the CBS2 or CBS3 domain, metformin treatment could still augment AMPKα phosphorylation at T172, though to a lesser extent. Furthermore, overexpression of the γ1 subunit with deletion of either the CBS1 or CBS4 domain led to a tendency for metformin-treated primary hepatocytes to produce more glucose than primary hepatocytes without metformin treatment. Since we found that metformin can bind to AMPK heterotrimeric complex^28^, and there are several glutamate residues within or around the CBS1 and CBS4 domains, from the functional point of view, it is possible that positively charged metformin can bind to the CBS1 and CBS4 domains, thus the CBS1 and CBS4 domains in the γ1 subunit may play important roles in metformin binding and AMPK activation. However, the metformin binding sites in the AMPK complex still remains to be determined.

## Materials and Methods

### Generation of adenovirus and adeno-associated virus

The BLOCK-iT adenoviral RNAi expression system (Invitrogen) was used to construct adenoviral shRNAs for γ1, γ2, and γ3 vectors as previously described^27,39,40^. The following sequences were used to generate shRNAs to effectively deplete γ subunit isoforms: γ1-2 (5′-GGTGGACATCTACTCCAAGTT-3′), γ1-3 (5′-CATCGGTCCCACTACT TTGA-3′); γ2-1 (5′-GCGTTTATATGCGATTCATGA-3′), γ2-2 (5′-GCAGGAGAACTTGAAC AAAGT-3′); γ3-1 (5′-CCCTCATCAAGAACCGAATC-3′), γ3-2 (5′-GGGCCTGAAATGCT TGGTTTC-3′). Subsequently, the vector of adenoviral shRNA for γ1 (γ1-3) was employed to generate AAV-vector. Regions in the pENTR/U6 vector containing the U6 promoter, Pol III terminator, and γ1-3 shRNA oligo or scrambled shRNA oligo were amplified by PCR and cloned into the AAV-BASIC vector (Vector Biolabs); these vectors were used to make AAV8 shRNAs for γ1 and scrambled shRNA. The adenoviral expression vectors of AMPKα1, α2, and β1 were generated as we reported previously^15^. To generate the AMPKγ1 mutants, FLAG-tagged γ1-WT, and γ1 mutants with deletion of individual CBS domains were subcloned into the pENTR2B vector (Invitrogen) and transferred into the pAd/CMV/V5-DEST vector (Invitrogen) by recombination to generate adenoviral expression clones^27^.

### Glucose production assays

Mouse primary hepatocytes were cultured in William’s medium E supplemented with ITS (BD Biosciences) and dexamethasone^15^. After 16 h of planting, the medium was changed to FBS-free DMEM for 3 h, and then, cells were washed twice with PBS, and the 1 mL glucose production medium (20 mM lactate, 2 mM lactate, pH7.4) was supplemented with vehicle or 10 nM glucagon. After 3 h incubation, both the medium and cells were collected. The medium was used to determine glucose concentrations with EnzyChrom Glucose Assay Kit^27^, and cell lysates were used to determine the protein levels in immunoblots.

### Animal experiments

All animal protocols were approved by the Institutional Animal Care and Use Committee of Johns Hopkins University and all animal experiments were carried out in accordance with relevant guidelines and regulations. To test the effect of AMPKγ1 on metformin suppression of liver glucose production, male C57BL/6 mice were fed an HFD (60% calories from fat) for 4 weeks, and mice were injected with AAV8 scrambled shRNA or γ1shRNA via the jugular vein (1 × 10^12^ GC/mouse). After treatment with metformin (50 mg/kg/day) for 3 weeks, a pyruvate tolerance test (16 h fast, 2 g/kg) was conducted.

### Cell cultures and immunoblots

24 h after the addition of adenoviral shRNAs, Hepa1–6 cells were exposed to metformin for 16–24 h before being harvested. Immunoblots were conducted as previously described^18,27^. Cell or liver lysates were homogenized and sonicated for 15 seconds three times and immunoblotted to examine the target proteins with antibodies against AMPKα1, α2, β1, γ1 (abcam) and pAMPKα (T172) (Cell Signaling) at the concentrations recommended by the manufacturers. Secondary antibodies were used at concentrations around 1:5000^41^.

### Indirect calorimetry

Male C57BL/6 mice were fed an HFD (60% calories from fat) for 4 weeks, followed by treatment with metformin for another 5 weeks. Mice weight gain and water consumption were measured every 7 days, and metformin concentrations in drinking water were adjusted accordingly. Mice were allowed to acclimate to respiratory chambers for 24 h. Oxygen consumption, carbon dioxide production, and heat production were measured for 48 h during 12 h light/12 dark cycles using the Comprehensive Lab Animal Monitoring System (CLAMS) (Columbus Instruments, Columbus, OH)^16^.

### Statistical analyses

Statistical significance was calculated with the Student’s t-test and ANOVA test. Significance was accepted at the level of p < 0.05. Sample size (number of mice) was determined on the basis of our previous studies^17,27^. At least 3 samples per group were chosen for statistically meaningful interpretation of results and differences in the studies using the Student’s t-test and analysis of variation.

## Supplementary information

Supplementary information.
