# Supplementary material for: The importance of the AMPK gamma 1 subunit in metformin suppression of liver glucose production
Source: Sci Rep. 2020 Jun 26;10:10482. doi: 10.1038/s41598-020-67030-5 (PMC7320014; doi:10.1038/s41598-020-67030-5)

The importance of the AMPK gamma 1 subunit in metformin suppression of liver glucose production

Hongying An[1], Yu Wang[1], Caolitao Qin[1,3], Mingsong Li[3], Akhil Maheshwari[1], Ling He[1,2]*

From the Departments of Pediatrics[1] and Pharmacology & Molecular Sciences[2], Johns Hopkins University School of Medicine, Baltimore, MD 21287, USA. Department of Hepatology[3], Southern Medical University, Guangzhou 510515, China.

*Correspondence should be addressed: Ling He, Departments of Pediatrics and Pharmacology & Molecular Sciences, Johns Hopkins University School of Medicine, Baltimore, MD 21287; Tel: 410-502-5765; Fax: 410-502-5779; Email: heling@jhmi.edu

Figure 1A

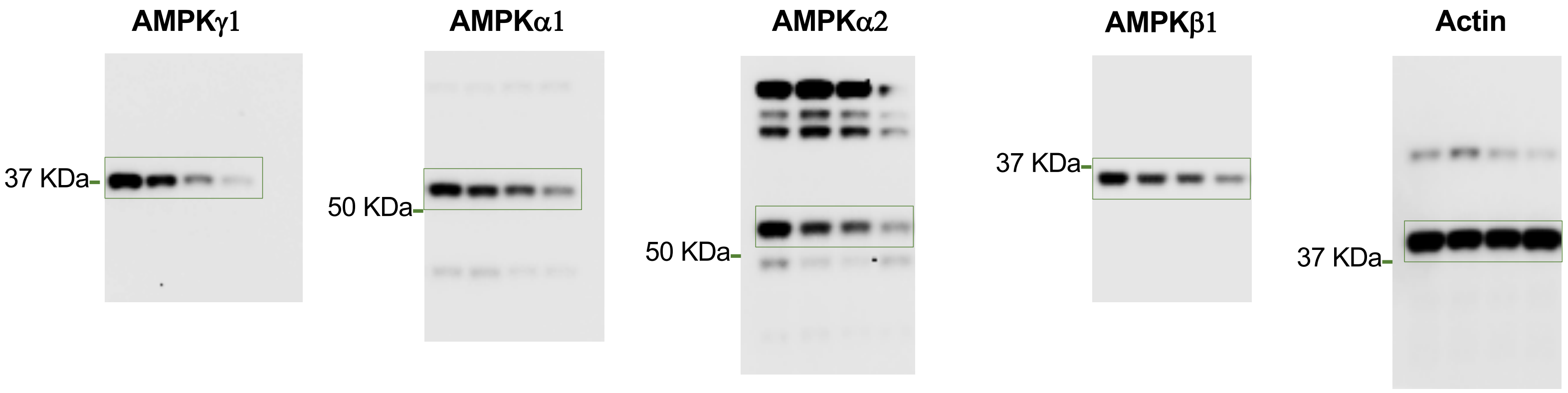

Figure 1C

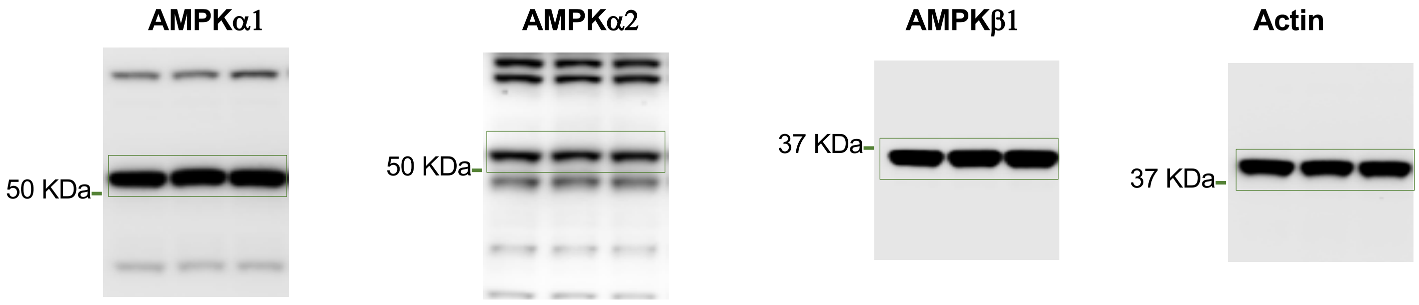

Figure 1E

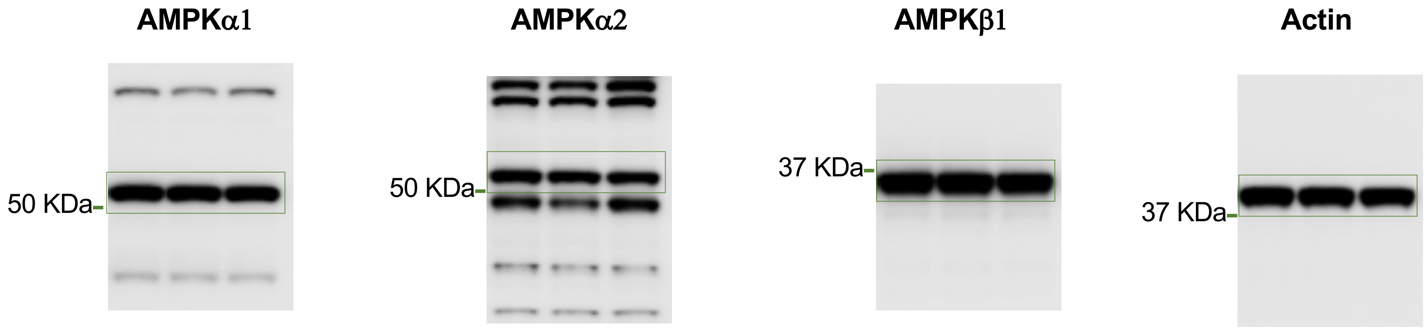

Figure 1G

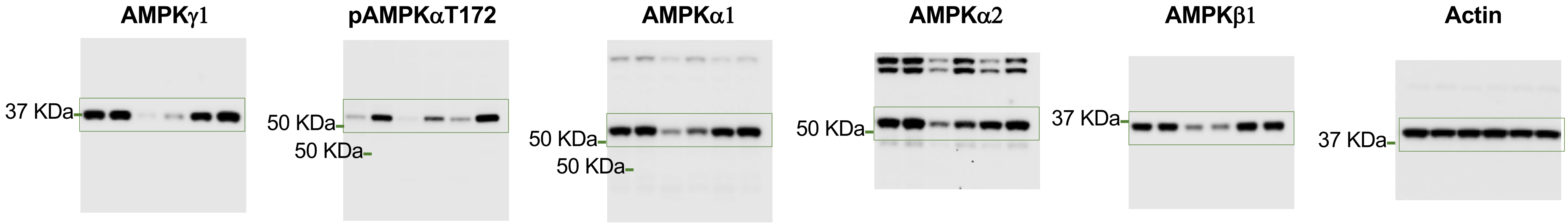

Figure 1H

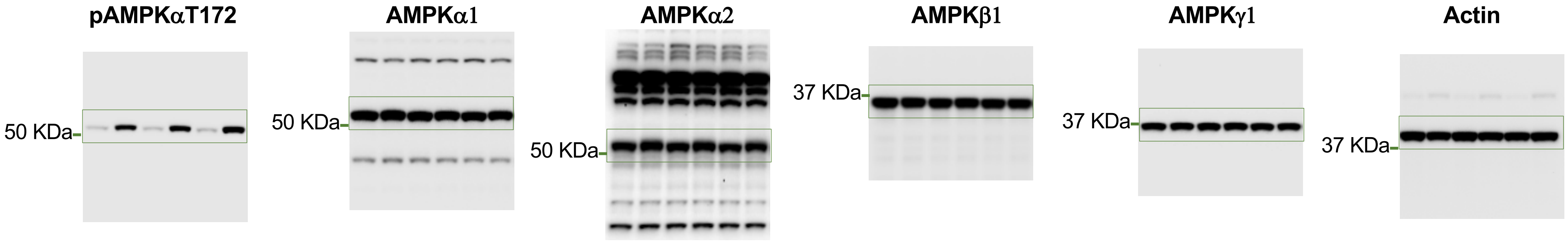

Figure 1I

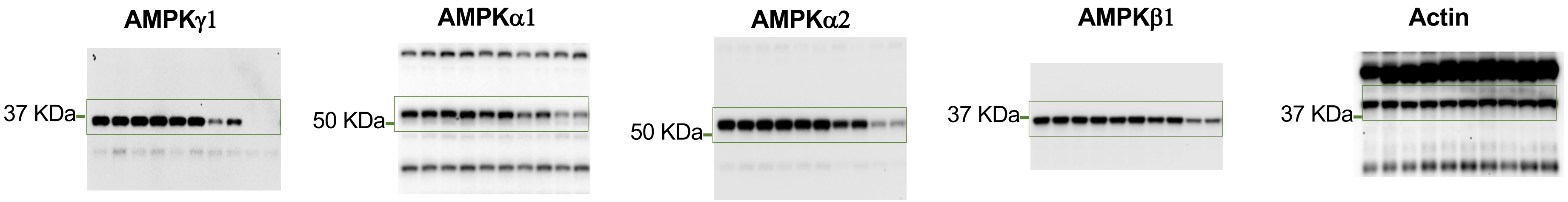

Figure 2A

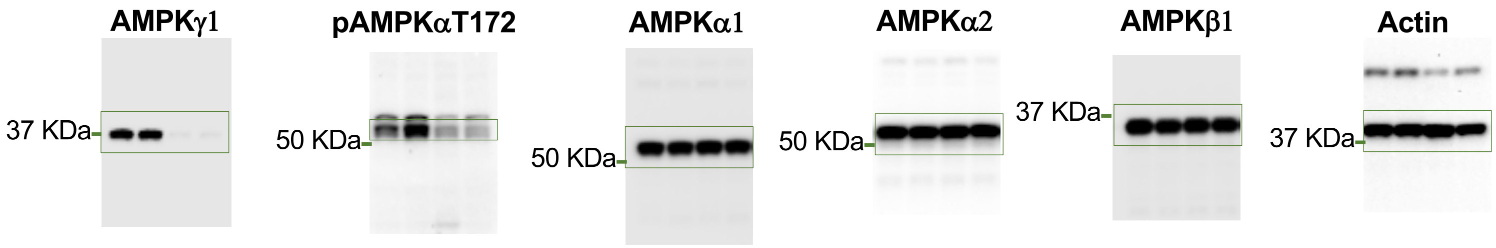

Figure 2B

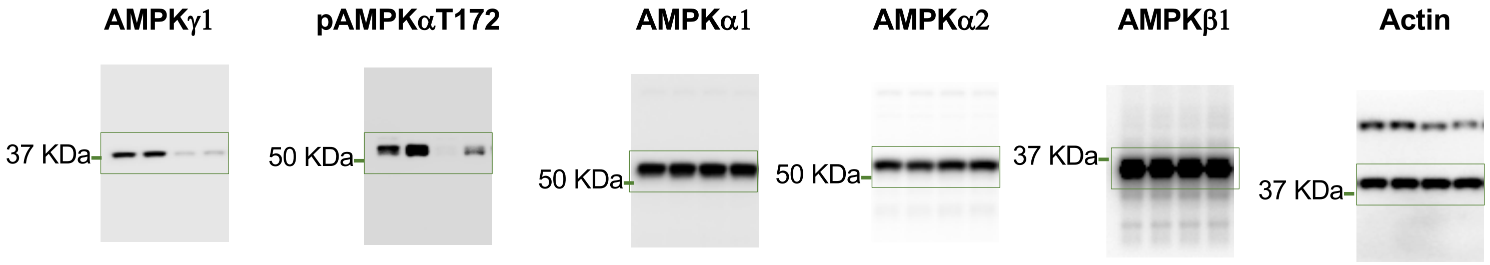

Figure 3B

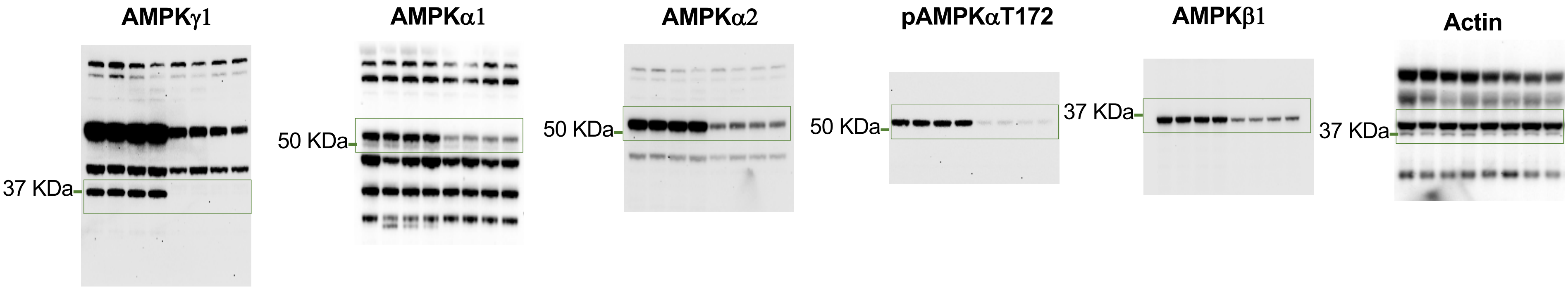

Figure 3F

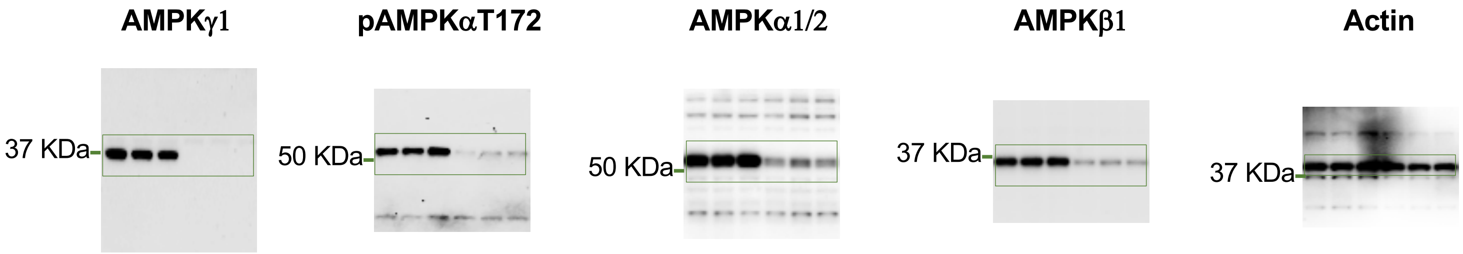

Figure 4C

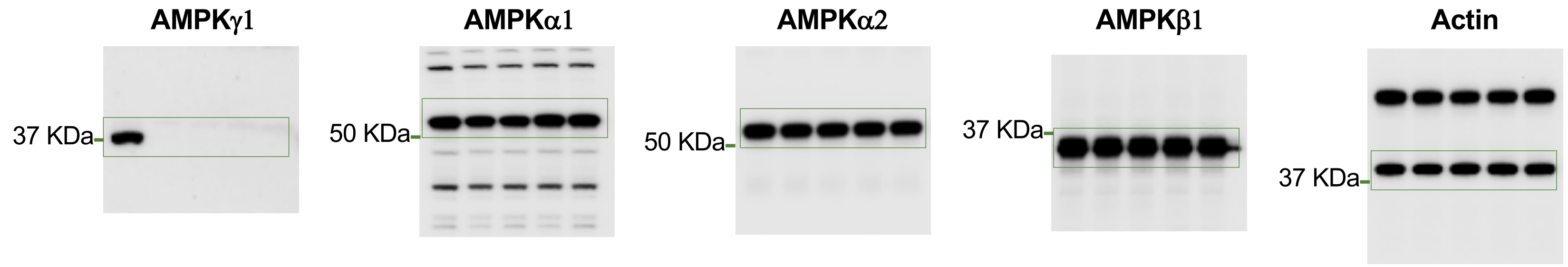

Figure 5B

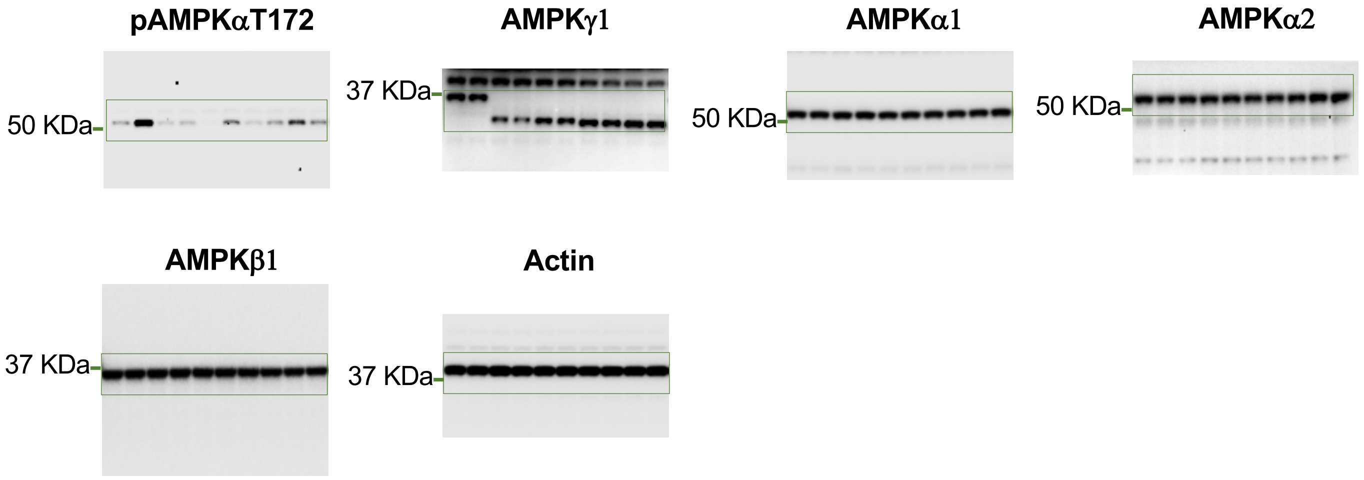

Supplement: Supplementary file 1 — Supplementary information. [file 41598_2020_67030_MOESM1_ESM.pdf]
